# Considerations of Autonomy in Guiding Decisions around the Feedback of Individual Genetic Research Results from Genomics Research: Expectations of and Preferences from Researchers in Botswana

**DOI:** 10.1155/2022/3245206

**Published:** 2022-03-31

**Authors:** Mary Kasule, Mogomotsi Matshaba, Erisa Mwaka, Ambroise Wonkam, Jantina de Vries

**Affiliations:** ^1^Botswana-Baylor Children's Clinical Centre of Excellence, Gaborone, Botswana; ^2^Baylor College of Medicine, Houston, TX, USA; ^3^College of Health Sciences, Makerere University, Kampala, Uganda; ^4^Department of Medicine, Faculty of Health Sciences, University of Cape Town, Cape Town 7925, South Africa; ^5^Deputy Dean's Office, Faculty of Health Sciences, Groote Schuur Hospital, Cape Town, South Africa

## Abstract

**Background:**

The Human Health and Heredity (H3Africa) Consortium continues to generate large amounts of genomic data leading to new insights into health and disease among African populations. This has however generated debate among stakeholders involved in developing, implementing, and applying ethical standards and policies for the return of individual genetic research results. The key questions are about when results must, should, may, or must not be returned and by whom. This study aimed to explore the views on the feedback of individual pertinent and incidental genetic research results of researchers, ethics committee members, and policymakers in Botswana.

**Methods:**

In-depth interviews were conducted with 16 key stakeholders from academic, research institutions, and regulatory bodies in Botswana. An analysis of the coded data was done through an iterative process of analytic induction to document and interpret themes and patterns.

**Results:**

Overall, the study indicated that researchers have at least a partial obligation to return individual genetic research results to research participants. Respondents placed emphasis on the ethical principle of autonomy. They felt that it was inappropriate for researchers to make decisions about the return of results on participants' behalf except in situations of avoiding participant self-harm or harm to society.

**Conclusion:**

Findings helped to highlight the importance of considering participants' autonomy in the development of sustainable and credible guidelines for feedback of findings from genomics research in Botswana, which can be explained during community engagement and consent processes. Such guidelines would ultimately be used to develop policies, guide African genomics research, and promote participant autonomy, transparency, and possibly participant trust in research.

## 1. Background

Genomic data generated from the Human Health and Heredity (H3Africa) Consortium is rapidly increasing [1, 2]. However, there continues to be scarce discussion around the feedback of individual genetic findings generated in the context of genomics research conducted in Africa [3]. H3Africa aims at facilitating research in genomics and environmental determinants of common diseases with the goal of improving the health of African populations [4, 5]. A deeper understanding of the data generated could arguably benefit participants in cases where results reveal a genetic predisposition to conditions that ought to receive medical attention or are preventable [3, 6]. This could also translate into a strong argument to afford research participants the opportunity to receive individual findings [7]. One important question in discussions around the feedback of individual genetic research findings generated in the context of genomics research relates to stakeholder expectations of and preferences for which findings are to be fed back, to and by whom, and when. Previously, the governing expert view discouraged the return of individual research results to participants because of the potential lack of analytic validity, questionable clinical validity, medical actionability, and questions about whether it is the role of the researcher to provide participants with their data [8]. With additional knowledge of participant perspectives and shifting views about the benefits of research and respect for participants, current expert consensus is moving toward the support of returning such results. Despite this recognition, significant ethical controversies remain especially the practical questions regarding appropriate procedures for returning results [1]. Another key ethical research challenge concerns what an investigator's obligations are if any to share information with those who volunteer to participate in his or her research. A debate has been ongoing elsewhere whether or not researchers are obliged to analyze and feedback to research participants' incidental findings and individual research results generated in genetic research [9]. Recently, similar debates have also been generated among African genomic researchers with the aim of developing guidelines for the feedback process [10]. Yet, there remains scarce published evidence from the African continent that could guide the feedback process. Internationally, authors have pointed to the importance of autonomy as a guiding ethical principle that could guide the process [11]. This is consistent with respect for persons and promotion of rational individual informed uncoerced decisions [12–14]. Furthermore, international guidelines recognize the need to respect and acknowledge research participants in advancing science [15], and some have argued that it is the researchers' duty to manage and offer the return of individual findings flow from the ethical duty of respecting participants' autonomy and rights [16]. Based at least in part on these recommendations, H3Africa developed a nonprescriptive set of guidelines which uphold autonomy as one of the criteria to follow [10].

Linked to the ethical principle of autonomy are the participants' right (not) to know and access health information, as well as investigators' fiduciary duties such as “duty to warn” and “duty to rescue” which could be used as a basis to determine the investigators' obligations in relation to the feedback of individual genomics research findings [17]. While a considerable amount of research has revealed how genomics researchers consider these various duties and values in North America [5, 8] and Europe [12] to date, there is a paucity of published evidence about how African investigators consider their obligations in return for individual genetic research results.

This paper culminated from one of the H3Africa Ethical, Legal, and Social Implications (ELSI) projects, namely, the Individual Findings in Genetic Research in Africa (IFGeneRA) study which aimed to explore the expectations and preferences of stakeholders involved in the conduct or and regulation of genomic research in Botswana. The study was part of a multicountry qualitative study conducted in three African countries: Botswana, South Africa, and Cameroon, under the auspices of the H3Africa Consortium but only results from Botswana are presented. The study was hosted by the Collaborative African Genomics Network (CAfGEN) coordinated at Botswana-Baylor Children's Clinical Center of Excellence which looked at a subset of HIV-positive children in Botswana to find out the host genetic factors that influence disease progression among pediatric Human Immunodeficiency Virus (HIV) and Tuberculosis (TB) infections that may account for some of the variability in the progression of this disease [18]. This study provided an anchor for our study as it has the potential of generating clinically important genetic results. In addition, Botswana has Standard Operating Procedures [19] for ethics review that have a section that mentions the return of results (7.2 section iv). This section states that, during the informed consent process, participants will be informed that the researchers will endeavor to provide information about the outcome of the research, will be advised when it is not intended to provide feedback, and will be asked whether or not they wish to be notified of research results that relate to them as individuals. A decision not to be notified should be respected. It is unclear whether this practice is practically followed. To find out more about the views of researchers, ethics committee members, and research regulators on whether and what individual genomic research results ought to be fed back and why, we conducted an empirical ethics study presented in this paper.

## 2. Methods

### 2.1. Setting and Study Design

The study was conducted in Gaborone, which is the capital city of Botswana, a Southern African country with a population of just over two million people. Most academic and research institutions are located in Gaborone. We conducted face-to-face, in-depth interviews with key stakeholders from academic research institutions and regulatory bodies involved in the conduct or regulation of genomic research in Botswana. Purposive sampling was used to identify key informants and generate rich data about stakeholders' expectations and preferences on the extent, nature, and timing of feedback of individual research findings including incidental findings of genomic research conducted in Botswana. The in-depth interview (IDI) guide that was used for data collection was developed by the IFGeneRA research team whose members had training and experience in conducting qualitative research studies on ethics-related topics in Botswana and other African countries. In addition, some of the team members who developed the IDI guide were experts in genetics and medicine. The interview guide was revised and verified by the lead researchers before use to ensure the appropriateness of the questions. The IDI contained short descriptions and examples to prompt in-depth discussions.

### 2.2. Study Population

This study involved stakeholders from academic research institutions and government ministries in Gaborone involved in developing, implementing, and applying ethical standards and policies for research involving human subjects research including genomics research. These included healthcare providers, ethics committee members, community advisory board members, researchers, and medical genetics professionals who had been involved in the conduct or regulation of genomics research for at least two years.

### 2.3. Sampling, Data Collection, and Analysis

Twenty-seven potential participants were invited to participate in the study and sixteen agreed to participate while nine did not respond to our invitation and 2 could not honor their appointments. Data was collected by the first author with assistance from a research assistant, both of whom are trained and experienced interviewers familiar with qualitative research methods. All participants were sent an invitation letter introducing them to the aims and objectives of the study as well as an Information Sheet. Each participant was also given a brief background of the researcher and the study prior to the commencement of the interview. The interviews were conducted in English at the respondents' workplaces and lasted approximately 45 to 60 minutes. All interviews were audio-recorded with permission from the respondents and were later transcribed verbatim. Interviews were conducted until saturation was reached [20], which we established in interim data analysis. No personal identifiers were collected, no master list was maintained that could link transcripts to respondents, and audio recordings were assigned study identification codes. Established procedures for qualitative research methods were followed to ensure rigor and trustworthiness of data collection, coding, and analysis procedures [21, 22]. Briefly, transcripts were first checked for accuracy and familiarization with data. Thematic analysis was conducted by two of the authors (MK and JDV), and the interview texts were then analyzed for content in line with the study aims. Guided by the objectives of the study, we initially open-coded selected transcripts to search for relevant concepts, and a hierarchical coding scheme was used to identify the main study themes and subthemes to generate a codebook. All transcripts were uploaded to and analyzed in NVivo qualitative Version 12 (QSR International Pty Ltd, 2012) software to aid in indexing, searching, and retrieving sections of data. In-depth analysis of the coded data was conducted through an iterative process of analytic induction to document and interpret themes and patterns.

## 3. Results

### 3.1. Demographics Characteristics of Study Population

Respondents' ages ranged between 41 and 60 years, and the majority were males (10/16). Important for this study was that respondents were highly experienced people in the fields of biomedical and social behavioral research with five having over 20 years of experience in this field of research. All respondents were highly qualified professionals with research training, with five at the Ph.D. level. Six respondents were medical doctors. Although most of the respondents were not directly involved in genomics research, having trained up to graduate level and beyond, they appeared to have solid background knowledge of genetics and heredity and its relation to health and disease, with eight trained in genetics at postgraduate level. Also, important for this study was the fact that respondents held administrative positions and key roles in biomedical and sociobehavioral research.

### 3.2. General Perspectives and Preferences on Autonomy and Respect for Persons

Broadly considering issues relating to the return of individual genetic research results, we found that respondents placed an important emphasis on autonomy and respect for persons as guiding values in thinking about the return of individual genetic findings to research participants. They also considered that these values translated into an obligation to return individual genetic research results, especially those that are actionable. Some respondents opined that all research results should be returned even if they are not actionable. Most respondents pointed out that participants have a “right to know” and should thus be allowed to decide what they would want to know and how they can use the information provided. Respondents felt that it would not be appropriate for researchers alone to make decisions on which results to feedback to research participants. One respondent who supported the return of all results said:

  IDI 04: “It's their information, it's not for you to tell somebody what or what not they need! You can explain to people, and you know the majority of people are poor! But, they understand when/if you take the time to explain things—so they can appreciate and understand what you are saying to them. Then let them decide for themselves, whether they want to hear or they do not want to hear!”

### 3.3. Investigator Obligations and Participant Rights

Some respondents questioned whether researchers indeed do have an obligation to feedback individual genetic research results, noting the challenges Botswana currently faces regarding the feedback process like lack of clear rationale or guidance for returning research to participants. However, one respondent who supported the researcher's obligation to feed back on the results described this nuance as follows:

  IDI 10: “Return findings from genetic tests that potentially bring up a finding where there is a direct clinical impact on that patient. Where there is something that can be done about the test results and would lead to a different patient outcome, I would prefer there was a mechanism in place by which you could return that result to the patient. I think that I would feel a strong desire or obligation to return that result back.”
  Although most respondents strongly felt they had an obligation to return all results as an obligation based on the participants” “right to know,” some respondents felt that the option of not disclosing based on the “right not to know” can sometimes be valuable, especially where disclosure could be potentially harmful (for instance because it creates anxiety) and should be respected. However, some respondents felt that it was important to equip participants with sufficient information to ensure understanding so that participants can make informed decisions. They particularly cautioned against researchers making decisions on behalf of participants, which they feared would lead to paternalism. There was a recommendation that in cases where a refusal to receive clearly actionable results would constitute self-harm, then the research team could disregard the participants' autonomy because they have a moral obligation of the “duty to warn” and “duty to rescue.” In such cases, respondents thought that researchers should make decisions that they felt were in the participants' best interest even if they were not aligned to the participants' wishes because sometimes this decision could be from a lack of understanding. Otherwise, individuals and society would stand to lose benefits from otherwise actionable results or results that could be actionable in the future. One respondent said:
  IDI 10: “I think it's important that participants should be made aware of a choice that might be detrimental for actionable results and those that have a direct implication to the participants and for transmission to other people (hereditary diseases) where there is loss of either benefit to the individual or to the society from actionable results. I see loopholes that the participants who say I'm not interested in getting the results it is not because they are not actionable but because of their understanding at that time! I always think that you need like in some cases a Medical Ethics Committee to look at this to evaluate self-injury in the legal context or in the ethical context because such injury could be driven by lack of understanding maybe lack of no knowledge of what alternatives are there at that time.”
  As a way of fulfilling participants' right to know or not to know, several respondents expected and preferred that issues of feedback of results should be included in the planning phase of study so that they can be discussed in the consent process and the plan should give participants the option of receiving results or not as a way of respect for persons. Like one of the respondents said:
  IDI 15: “It's the responsibility of the researcher in the planning phase of the research to scope all the possibilities of the incidental findings and preplan how to deal with them. The research protocol submitted for IRB review should have a plan for the return of findings.”
  Some respondents, however, argued that despite the importance of upholding autonomy, an outright promise of feedback of findings or asking participants what results they would like to receive could be a challenge due to the current uncertainty about the clinical meaning of some results. Like one respondent said:
  IDI 10: “Do not feedback because genetic information that is not clearly known yet. Feedback should be limited to things that are very established predictors being used in clinical testing already. You might find there are things that they [participants] would want to get back but you don't fully understand what a lot of these things mean yet. I don't see how you could do that really in a way that would be meaningful or understandable or that would have any impact on their future health or well-being. This would also have the strong potential to cause anxiety and confusion.”

### 3.4. Autonomy and Complexity of Genomics Information

Considering the complexity of genomic information, some respondents expressed skepticism about the possibility of upholding autonomy and respect for persons as challenges may arise from communicating complex genomic information such as genomic risk predictions in numerical probabilities. One respondent described this challenge as follows:

  IDI 10: “We don't know how big a risk some of the things are and aren't so it's very difficult to know at what point it becomes established enough to feel that you can tell people that this is in fact, a risk. Communicating risk is very difficult to people anyway, you know telling people that there is 10 times the risk of something sounds terrible, but if the risk was, you know, just a thousand of a thousandth of a percent in the first place is still a small risk.”
  Most respondents noted that the majority of research participants in Botswana usually have low levels of education and genomic literacy which can potentially compromise understanding during the consent process. For this reason, all respondents emphasized the importance of educating people directly affected by or involved in genomics research as essential to promoting autonomous choices. This education was considered as a form of empowerment that could improve participants' understanding of the information provided, demystify genomics, avoid misinformation, promote transparency on how samples are used, and enable valid informed decisions. One of the respondents described the importance of education as follows:
  IDI 14: “Ya, I think ah,—maybe feeding back of information, like I was saying, depends on how people are knowledgeable about what you are doing. For us [referring to Botswana setting) we don't read, we are being told. So, we are not well informed, even if it might be good to be told about a lethal condition, the fact that I don't understand and know the consequences and where to get information or why did they tell me something like this, if they don't have treatment. So, it gives a different perspective.”
  Regarding the question of who should return the results, another form of promoting autonomous decision-making by participants that was suggested by some of the respondents was providing genetic counseling. However, one respondent who had trained in psychology and counseling felt that currently Botswana is not in an ideal position to do genetic counseling due to a lack of experts in this field. This was expressed as indicated below:
  IDI 13: “I feel there is no genetic counseling in Botswana because genetic counseling is complex, it is a skill that not everybody has including myself who has trained in psychology and counseling and I cannot engage with someone who has a genetic problem. I think we do not counselors with the right qualities to counsel people with genetic problems. What is currently done is so mechanical, so we need to build a lot of capacity in this area.”

### 3.5. Autonomy and Which Results to Be Feedback

Some respondents expressed concern about the kind of results that should be returned. While some respondents strongly felt that all results should be returned, some emphasized that much as it is very important to respect participants' right to self-determination, it is important to return only results that are verified and actionable. They posited that these issues should be considered in community engagement activities and the informed consent process. International guidance demands that only clinically significant findings that have been diagnostically validated by an accredited laboratory should be fed back to research participants [17]. However, respondents felt that diagnostic validation has cost implications which even Botswana government might not afford despite health care being free for all citizens. One respondent expressed this concern as follows:

  IDI 15: “You need to give those [results] that are confirmed from an accredited laboratory.—But at whose cost? Because it is really expensive, we are talking about thousands of Pula [Hundreds of dollars). Now before I leave that one, in Botswana we have this mentality that health care is free, right? So you've got these results. You have confirmed them, where next—where do you send the participant because even government might not be able to afford some of these interventions and government has got other pressing needs like TB.”
  While the importance of embedding discussions in an engagement format was a recurring theme, the suggestion that perhaps an independent third party could play a role in deciding on feedback, where there is an obvious clinical benefit on the one hand and a refusal to receive information on the other, was also recurring.
  IDI 09: “it's just that now there needs to be a third party that looks beyond, that evaluates self-injury in the context, in the legal context or in the ethical context because such injury could be driven by lack of understanding maybe lack of no knowledge of what alternatives are there at that point in time.”
  However, going through a third party could be complicated by issues of breach of privacy and confidentiality and viewed as being paternalistic. Another respondent advised that researchers must be transparent and honest by not hiding fundamental information from the participant even if they opted not to know.
  IDI 03: “I think we always owe it to people to be that transparent because there's a lot that is done in the name of good intentions, but the lines get blurred too much—because you know, people's boundaries can be overstepped”.

## 4. Discussion

In this manuscript, we drew attention to the emphasis placed by persons involved in the conduct or regulation of genomics research in Botswana, on autonomy and respect for persons in making decisions on the return of individual genetic research results. Consistent with other scholars, respondents considered that these values should be primary in deciding which individual genetic research results should be fed back in genomics research. This is in line with the H3Africa consensus on the need for participant volition as one of the criteria that needs to be met for feedback of individual genetic results. [10, 17]. Similarly, this is also recognized in international guidelines [12, 14, 23]. However, some respondents suggested that respecting autonomy does not translate into an obligation to return all results but, rather, the need to balance autonomy with other considerations such as the clinical utility of the findings. Equally important is the availability of resources to safely return actionable results to participants.

We outlined that our findings suggested that going forward, researchers in Botswana need to plan for feedback of findings. They could use the list of variants that have already been validated [24] to develop lists of (kinds of) research results specifically for Botswana that ought to be considered for feedback *prior to* the start of the research projects. Developing these lists would heavily depend on the availability of diagnostic testing facilities to validate the findings which could pose a challenge. Furthermore, once such lists are developed, our respondents indicated that it would be very important to include information about the possible return of individual genetic research results in the consent process, where participants are specifically asked whether they would like to receive such results should they be available. Our respondents placed a lot of emphasis on the process of community engagement as one way to empower participants and to promote autonomous choice. It is possible that engagement activities should also lie at the basis of determining the most appropriate ways of relaying the results back to individuals, families, and the community. Engagement activities could also assist in the development of appropriate educational strategies to promote understanding of genomics and issues around the return of individual genetic research results, both of which are essential to promoting autonomous decision-making by prospective participants. Community engagement has been recognized as a way of extending the ethical principle of autonomy and respect for persons to the entire community which promotes trust and researcher-participant relationships, thereby protecting against exploitation [25]. Furthermore, in the South African context and many other parts of Africa including Botswana, the concept of “*Ubuntu*” is a component of responsive communitarianism where communal welfare is valued together with individual autonomy [26]. Although the Botswana Ministry of Health and Wellness has set up Community Advisory Boards in various districts, a number of respondents recommended the need for community empowerment specific to genomics research and genomics literacy which they thought were lacking. This would promote understanding and ease participant recruitment into genomics studies. It would also lead to community buy-in, foster trust, and transparency as well as enrich participant-researcher relationships.

Participants should be free to decide whether or not to receive results even if they choose not to receive them. Our respondents indicated that only in some situations, namely, those where not receiving results would constitute “self-harm,” could such an autonomous choice be trumped. However, our respondents failed to clearly describe what would constitute “self-harm.” Respondents also recommended genetic counseling as a form of promoting participants' autonomy; however, they also expressed concern about the lack of expertise in this field in Botswana and the need for capacity building.

### 4.1. Limitations of the Study

A limitation of our study is that we only spoke to older interviewees whose views may have been influenced by their age or their relative professional seniority. Another limitation is that this study was conducted in Botswana, which is a relatively wealthy middle-income African country, with a small population and where many research participants are likely to have access to actionable medical interventions because of the availability of Universal Health Care. Therefore, practical considerations of actionability were not a factor in the views articulated by our participants. If this study were conducted in an environment where there were severe resource restrictions and healthcare limitations, the respondents would possibly have given greater prominence to such considerations. Furthermore, our relatively small sample size might not be a large enough sample to sufficiently describe and address the research question at hand; hence, these findings may not be generalized.

## 5. Conclusion

Overall, our study indicated that researchers in Botswana have at least a partial obligation to return individual genetic research results to participants, with great emphasis placed on processes that promote autonomous choice. Consequently, autonomy could be considered as a guide to decisions surrounding the return of individual genomic findings. Our respondents did not seem to think it appropriate for researchers to make decisions about the return of results on participants' behalf, provided that the information that would be shared was trustworthy (meaning, scientifically valid, clinically meaningful, and validated), except in situations where researchers are avoiding self-harm. Going forward, we recommend that greater attention is given to the development of sustainable and credible feedback of findings policies in genomics research in Botswana, which can be explained during community engagement and consent processes. Such policies would improve transparency, promote participant autonomy, and possibly promote participant trust in research.

## Data Availability

The datasets used and/or analyzed during the current study are available from the corresponding author on request using the e-mail address provided.
